# *BmC/EBPZ* gene is essential for the larval growth and development of silkworm, *Bombyx mori*

**DOI:** 10.3389/fphys.2024.1298869

**Published:** 2024-03-07

**Authors:** Xinglin Mei, Tianchen Huang, Anli Chen, Weibin Liu, Li Jiang, Shanshan Zhong, Dongxu Shen, Peitong Qiao, Qiaoling Zhao

**Affiliations:** ^1^ College of Biotechnology, Jiangsu University of Science and Technology, Zhenjiang, Jiangsu, China; ^2^ Key Sericultural Laboratory of Shaanxi, Ankang University, Ankang, Shaanxi, China; ^3^ Sericultural Research Institute, Chinese Academy of Agricultural Sciences, Zhenjiang, Jiangsu, China

**Keywords:** *Bombyx mori*, *BmC/EBPZ*, *GMS* mutant, small larvae, positional cloning, CRISPR/Cas9

## Abstract

The genetic male sterile line (*GMS*) of the silkworm *Bombyx mori* is a recessive mutant that is naturally mutated from the wild-type *898WB* strain. One of the major characteristics of the *GMS* mutant is its small larvae. Through positional cloning, candidate genes for the *GMS* mutant were located in a region approximately 800.5 kb long on the 24th linkage group of the silkworm. One of the genes was *Bombyx mori* CCAAT/enhancer-binding protein zeta (*BmC/EBPZ*), which is a member of the basic region-leucine zipper transcription factor family. Compared with the wild-type *898WB* strain, the *GMS* mutant features a 9 bp insertion in the 3′end of open reading frame sequence of *BmC/EBPZ* gene. Moreover, the high expression level of the *BmC/EBPZ* gene in the testis suggests that the gene is involved in the regulation of reproduction-related genes. Using the CRISPR/Cas9-mediated knockout system, we found that the *BmC/EBPZ* knockout strains had the same phenotypes as the *GMS* mutant, that is, the larvae were small. However, the larvae of *BmC/EBPZ* knockout strains died during the development of the third instar. Therefore, the *BmC/EBPZ* gene was identified as the major gene responsible for *GMS* mutation.

## Introduction

CCAAT/enhancer-binding proteins (C/EBPs) belong to the basic region-leucine zipper (b-ZIP) protein family (Ramji and Foka, 2002). C/EBPs were discovered as heat-stable proteins in rat liver nuclei (Johnson and McKnight, 1989). To date, six members of the C/EBP family have been identified: C/EBP α, C/EBP β, C/EBP γ, C/EBP δ, C/EBP ε and C/EBP ζ (Ramji and Foka, 2002). C/EBPs typically has three structural components: a C-terminal leucine zipper domain, DNA-binding domain, and N-terminal transcriptional activation domain (Tenen, 2003). C/EBP factors are mainly involved in cell growth and differentiation, immune response, inflammatory response, energy metabolism, tumorigenesis, and apoptosis and regulate the transcription of target genes (Nerlov, 2007; Jin et al., 2011). C/EBP α is the first described C/EBP protein and plays an important role in many cellular responses and adipocyte and hepatocyte differentiation (Lefterova et al., 2008; Li et al., 2010; Pang et al., 2021; Chowdhury et al., 2023). The first discovered C/EBP β was NF-IL6, which plays an important role in many cellular processes, such as cell differentiation, tumorigenesis, apoptosis, energy metabolism, and reproduction (Akira et al., 1990; Ghani et al., 2022). C/EBPs contain an N-terminal transcriptional activation domain, except C/EBP γ. Mice deficient in the C/EBP γ gene have high post-birth mortality rates (Lekstrom-Himes and Xanthopoulos, 1998; Kaisho et al., 1999; Renfro et al., 2022). The expression level of C/EBP δ in human and adult mouse tissues is extremely low or even undetectable. C/EBP δ not only can promote inflammatory signals but also can inhibit pro-inflammatory pathways. However, despite that it can reduce the incidence of tumors, it promotes tumor metastasis (Balamurugan and Sterneck, 2013). C/EBP ε plays a vital role in terminal neutrophil differentiation. Mice deficient in C/EBP ε exhibit abnormal terminal granulocytic differentiation and typically succumb to bacterial infections within 5 months. (Tavor et al., 2002). A C/EBP ζ named DNA damage-inducible transcript 3 is associated with the development of leukemia, melanoma, and myxoid liposarcoma (Wang et al., 2011).

C/EBPs are present in various species, including mice, humans, chickens, *Xenopus laevis*, *Rana catesbeiana*, and fish (Johnson and McKnight, 1989; Akira et al., 1990; Roman et al., 1990; Cao et al., 1991; Ron and Habener, 1992; Hartl et al., 2023). The C/EBP DmC/EBP was first reported in insects (*Drosophila*) in 1992 (Rørth and Montell, 1992). It plays an important role in embryonic development. C/EBP factors regulate the expression of ferritin heavy-chain genes in *Aedes aegypti* and sterol carrier genes in *Spodoptera litura* (Pham et al., 2005; Liang et al., 2015). In 2005, the C/EBP BmC/EBP was discovered in silkworm (Sourmeli et al., 2005). It was the first C/EBP discovered in lepidopterans. In silkworm, the *BmC/EBPg* gene regulates histone acetylation and the expression of chorion gene (Lyu et al., 2020).

The genetic male sterile silkworm line (*GMS*) is naturally mutated from the wild-type *898WB* strain. One of the major characteristics of the *GMS* mutant is that the larvae are small (Supplementary Figure S1). The wild-type *898WB* were reared at normal temperature and humidity standards, and *GMS* mutants appeared in eight moth areas when the larvae developed to the 2nd instar. The silkworm larvae in eight moth areas were observed, and the separation ratio between the normal and mutant individuals was approximately 3:1. The results showed that the *GMS* mutant trait was heritable and controlled by a recessive single gene. RNA-Seq results have identified many differentially expressed genes between the *GMS* mutant and wild-type *898WB*, such as immune-related genes, serine or threonine protein kinase-encoding genes, and heat shock protein genes (Mei et al., 2022). In the present study, positional cloning and gene sequencing showed that the *BmC/EBPZ* gene is the major gene responsible for *GMS* mutation. We further verified the role of the gene in the growth and development of silkworm by using the CRISPR/Cas9-mediated knockout system. Further analysis of the important role of *BmC/EBPZ* gene in the silkworm can help to provide potential targets for pest control.

## Materials and methods

### Silkworm strains

The *GMS* mutant, wild-type *898WB*, *Nistari*, and *Dazao* (P50) silkworm strains were maintained in the key laboratory of Sericultural Biology (Sericultural Research Institute of the Chinese Academy of Agricultural Science, Jiangsu, China). The larvae were fed with mulberry leaves and reared at 25°C ± 1°C and 65% ± 5% relative humidity.

### Materials used for positional cloning

The silkworm *GMS* mutant (gms/gms) mated with the p50 strain (+^gms^/+^gms^) to generate F_1_ offspring. Then, the female F_1_ and male F_1_ offspring were backcrossed with the *GMS* mutant to produce BC_1_F (♀F_1_×♂*GMS*) and BC_1_M (♀*GMS*×♂F_1_) populations, respectively. The *GMS* mutant, F_1_ offspring, and p50 can be used to screen polymorphic genetic markers. No genetic exchange was observed during meiosis in the female silkworms. The BC_1_F populations (10 wild-type strains and 10 mutant strains) were used for screening the linkage group, and the BC_1_M populations (252 mutant strains) were used for constructing the molecular marker linkage map of the mutant gene.

### DNA, RNA extraction, and qRT–PCR

The heads of fifth instar larvae were used in genomic DNA extraction. First, a DNA extraction buffer (50 mM Tris-cl [pH 8.0], 10 mM EDTA [pH 8.0], 50 mM NaCl, 0.1% SDS, 20 mM proteinase K) was mixed with the tissues. DNA was then purified with phenol and chloroform and washed with 75% ethanol. Then, genomic DNA was precipitated with anhydrous ethanol. Finally, the DNA was dissolved in ultrapure water and diluted to 50 ng/μL. Total RNA extraction was conducted according to our previously described method (Mei et al., 2022).

Differences in gene expression levels between the *GMS* mutant and wild-type *898WB* were determined through qRT–PCR. The total volume of the qRT–PCR reaction was 20 μL, composed of 1 μL of primer (10 μM), 1 μL of EvaGreen (Biotum, United States), 1 μL of total RNA (50 ng/μL), 10 μL of 2× one-step RT–PCR Mix (XT Biotech, China), and 7 μL of ultrapure water. The silkworm housekeeping gene (*BmGAPDH*) was used to normalize the qRT-PCR results. A QuantStudio 3 system (Thermo Fisher, United States) was used for qRT–PCR. The PCR amplification program was as follows: 50°C for 30 min; 95°C for 1 min; and 45 cycles of 95°C for 15 s and 58°C for 30 s. The qRT-PCR reaction was repeated three times for each sample, and gene expression levels were analyzed by the 2^−ΔΔCT^ method. The significance of difference was analyzed by two-tailed *t*-test (*p < 0.05, **p < 0.01, ***p < 0.001).

### Preparation of the genetic linkage map

The simple sequence repeat (SSR) genetic markers were used for screening the linkage group and constructing a genetic linkage map. The genome sequences of the 28 linkage groups of silkworms were downloaded from SilkDB 3.0 (https://silkdb.bioinfotoolkits.net/). The primers were designed using the software Primer Premier 5.0 and synthesized by BGI (Beijing, China). A mutant gene was considered to be on the same chromosome with the SSR marker when the DNA typing of wild-type individuals in the BC_1_F populations were the same as that of the F_1_ offspring or *GMS* mutant parental strain and the DNA typing of mutant individuals was the same as that of the *GMS* mutant parental strain. After the linkage group was confirmed, other polymorphic SSR molecular markers on the linkage group were used in the construction of a linkage map, and the location of a candidate gene was finally confirmed according to the number of individuals that showed genetic exchange.

### Candidate genes analysis

On the basis of the results of fine mapping, the silkworm database SilkDB 3.0 (https://silkdb.bioinfotoolkits.net/) and KAIKObase (https://kaikobase.dna.affrc.go.jp/) were used in the screening of genes located in the linkage group. The open reading frame (ORF) sequences of the candidate genes were downloaded. The structures and expression levels of the candidate genes were analyzed on the basis of the results of RNA-Seq (Mei et al., 2022) and qRT–PCR. Differences in candidate genes between the *GMS* mutant and wild-type *898WB* were identified to confirm the gene that was mainly responsible for *GMS* mutation. The SMART database (http://smart.embl-heidelberg.de/) was used to predict the domain of the candidate gene, and Mega7.0 was used to construct the phylogenetic tree by Neighbor-Joining method, and other parameters were the default values of the software.

### CRISPR/Cas9-mediated knockout

According to the 5ʹ-GGNN18NGG-3ʹ design rule of CRISPR/Cas9 target, two single-guide RNA (sgRNA) sites in the ORF region of *BmC/EBPZ* gene were designed by the online software CRISPR direct (http://crispr.dbcls.jp/). The DNA sequence containing the two targets of sgRNA1 and sgRNA2 of the *BmC/EBPZ* gene was amplified. We ensured whether the two sgRNA sites of the genomic DNA of the *Nistari* strain had mutations by cloning and sequencing to prevent CRISPR/Cas9 off-target. Two pairs of primers containing sgRNA sites were used to amplify the plasmids constructed in our laboratory, and then the sgRNA sequence was homologously recombined with the initial plasmid (pXL [IE1-DsRed-U6]) to produce transgenic plasmids containing sgRNA sites (pXL [IE1-DsRed-U6-sgRNA]). The sgRNA transgenic plasmid contains a *DsRed* gene that encodes a red fluorescent protein and two sgRNA sites. The *DsRed* gene was initiated by, I.E.,1, and the sgRNA was initiated by U6 promoter. The primers were designed according to the recombinant plasmid, and plasmid integrity was verified by cloning and sequencing.

*Nistari* fertilized eggs were prepared, and then the sgRNA transgenic plasmid was mixed with the *PiggyBac* helper plasmid and microinjected into the prepared silkworm eggs. The eggs were regarded as the G_0_ generation, and then the adults of the G_0_ generation self-mated. The sgRNA-positive silkworms that expressed red fluorescent proteins were screened under a fluorescence microscope (Nikon AZ100, Japan), and the G_1_-positive individuals were mated with the Cas9 transgenic silkworms expressing green fluorescent protein. Finally, double-positive silkworms that expressed both fluorescence proteins were screened in the G_2_ generation, which were the knockout strains.

Three mutant individuals were randomly selected and genomic DNA was extracted. PCR amplification was performed using cloned primers containing two sgRNA sites, and the amplified sequence was cloned into pMD18-T vector (TaKaRa, China) for sequencing verification. Finally, it was determined whether the target gene was successfully edited by the CRISPR/Cas9 system.

## Results

### Candidate genes of the *GMS* mutant were located on the 24th linkage group

**FIGURE 1:**
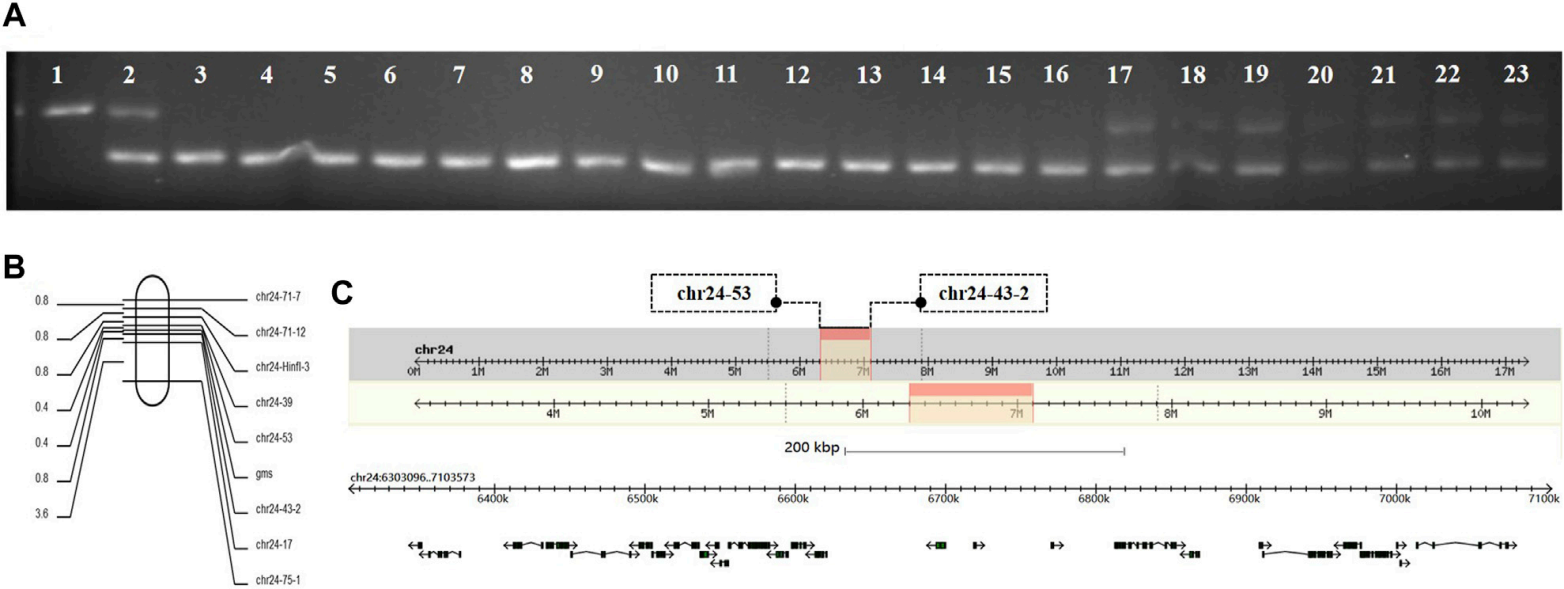
Genetic linkage group analysis of *GMS* candidate genes. **(A)** Lanes 1, 2, and 3 are the parent strain P50, BC_1_F offspring, and *GMS* mutant parent, respectively. Lanes 4–13 are BC_1_F populations with mutant phenotypes. Lanes 14–23 are BC_1_F populations with wild-type phenotypes. **(B)** Genetic linkage map between the *GMS* candidate gene and polymorphic molecular markers. **(C)** The physical map of the *GMS* candidate gene located on the 24th linkage group. The black box with arrows represents the 27 candidate genes.

Genomic DNA was extracted from the *GMS* mutant, *P50*, and BC_1_F individuals, and the extracted DNA was amplified by SSR primers for the screening of polymorphic molecular markers. The genomic DNA of 10 mutant and 10 wild-type individuals in the same batch of BC_1_F populations were extracted. The SSR primers with polymorphisms were used for PCR amplification and verification of the linkage relationship between the SSR polymorphic markers and *GMS* mutant genes. When PCR amplification was performed with polymorphic marker primer chr24-71-7 on the 24th linkage group of silkworms, the results showed that the DNA typing of mutant individuals in the BC_1_F populations were the same as that of the *GMS* mutant parental strain, and the DNA typing of wild-type individuals was the same as that of F_1_ offspring or *GMS* mutant parental strain (Figure 1A). The result indicated that the *GMS* mutant gene was located on the 24th linkage group of silkworms. The sequences of the primer chr24-71-7 are listed in Supplementary Table S1.

### Construction of the SSR molecular marker genetic linkage map

To determine the accurate location of the *GMS* mutant gene on the 24th linkage group, we used 252 BC_1_M mutants for genotype analysis. The genomic DNA of each mutant was used as a template, and primers for PCR amplification was performed using all the SSR polymorphic markers on the 24th linkage group (Supplementary Table S1). The DNA typing of mutants in the BC_1_M populations should be the same as that of the *GMS* mutant parental strain. When the *GMS* mutant gene of the F_1_ male parent showed genetic exchange with the SSR marker, the DNA typing of mutant individuals in the BC_1_M populations was be the same as that of the F_1_ offspring. According to the results of genotype analysis, a genetic linkage map of the molecular markers was drawn by Mapdraw software (Figure 1B). The mutant gene was located between polymorphic markers chr24–53 and chr24-43-2. The genetic distance between the two markers was 7.54 cM, and the physical distance was 800.5 kb (Figure 1C).

### *BmC/EBPZ* gene was identified as the major gene responsible for *GMS* mutation

**FIGURE 2:**
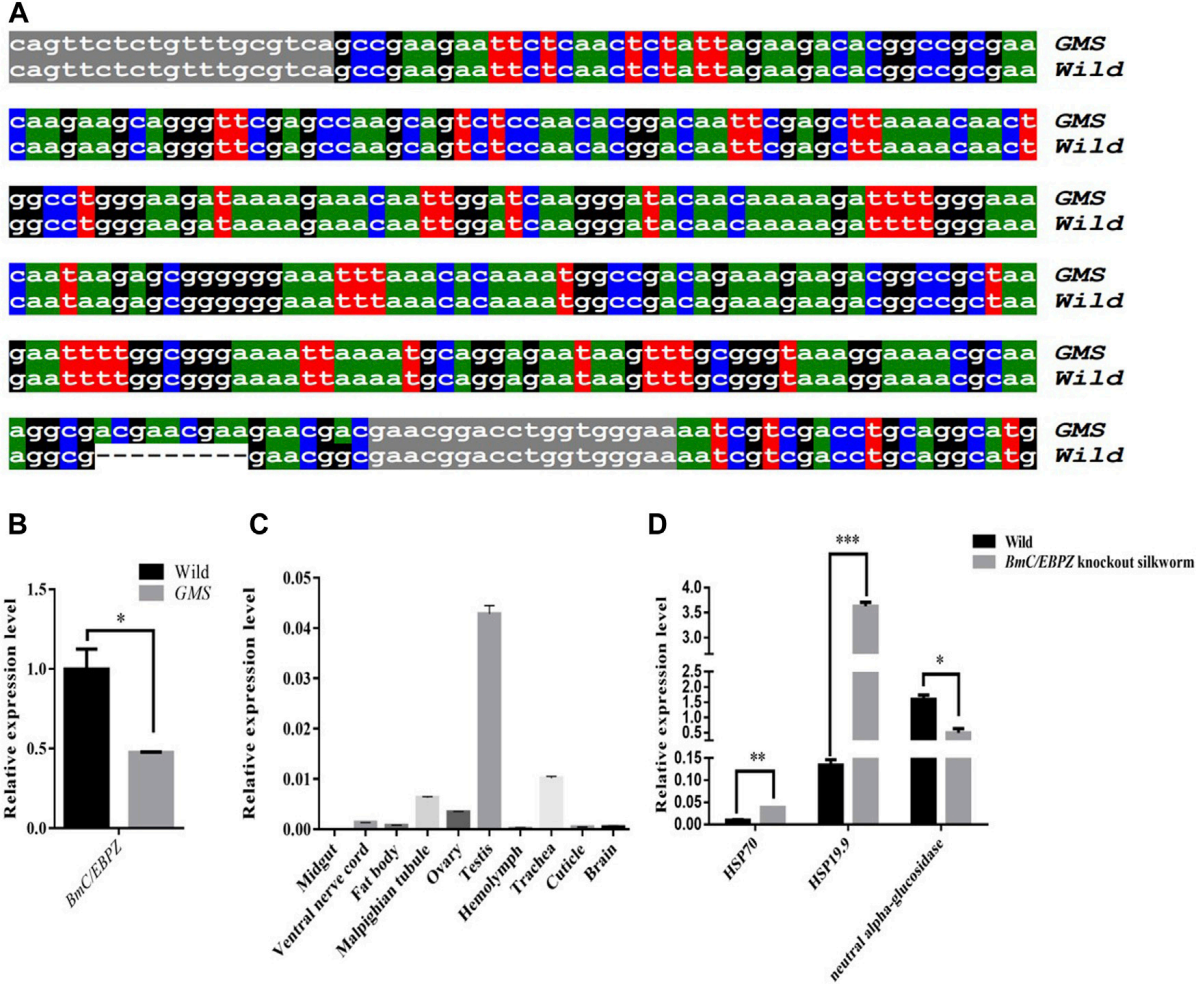
Sequencing results and relative expression analysis of the *BmC/EBPZ* gene. **(A)** Sequence alignment of the partial ORF of *BmC/EBPZ* gene between the *GMS* mutant and wild-type *898WB*. Gray marker sequences indicate the position of the cloning primer. “---” indicates that the sequence deletion. **(B)** The relative expression levels of the *BmC/EBPZ* gene between the *GMS* mutant and wild-type *898WB*. **(C)** The relative expression levels of the *BmC/EBPZ* gene in different tissues of P50. **(D)** The relative expression levels of *HSP70*, *HSP19.9* and *neutral alpha-glucosidase* between the *BmC/EBPZ* gene knockout strain and wild-type *898WB*.

Fine mapping results showed that the mutant gene was located between two polymorphic markers chr24–53 and chr24-43-2 on the 24th linkage group, and this specific region contained 27 annotated genes (Supplementary Table S2). According to our transcriptome sequencing results (Mei et al., 2022), the expression levels of and structural variations in the candidate genes were analyzed. Then, on the basis of the RNA-Seq and Sanger sequencing results, a 9 bp-long sequence was inserted into the 3′end of the ORF sequence of the *BmC/EBPZ* gene in the *GMS* mutant (Figure 2A). The qRT–PCR results showed that the *BmC/EBPZ* gene was differentially expressed between the *GMS* mutant and wild-type *898WB* (Figure 2B). The relative expression levels of the *BmC/EBPZ* gene in the different tissues of the wild-type strain (P50) were analyzed. The results showed that the expression level of *BmC/EBPZ* gene was higher in the testis, trachea, malpighian tubule, ovary, lower epidermis, fat body, ventral nerve cord, and brain, but nearly no expression was observed in the midgut (Figure 2C). The above data indicated that the *BmC/EBPZ* gene was the major gene mainly responsible for *GMS* mutation. The qRT–PCR primers are listed in Supplementary Table S3.

### CRISPR/Cas9-mediated knockout of the *BmC/EBPZ* gene

**FIGURE 3:**
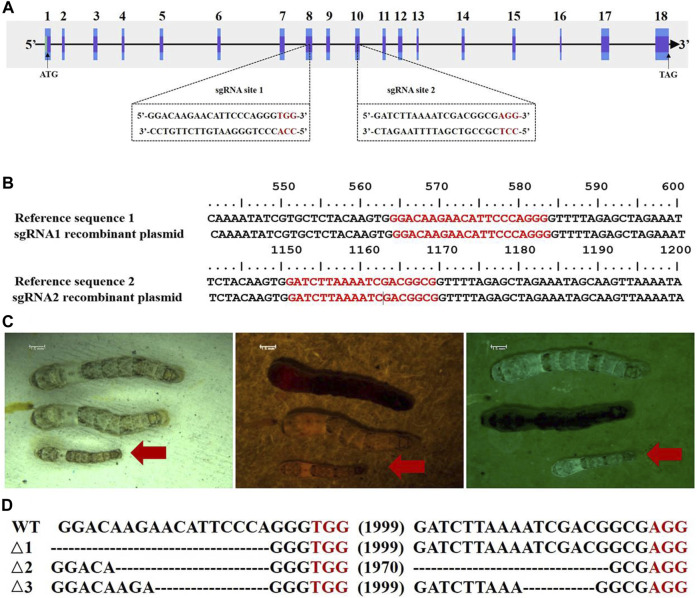
CRISPR/Cas9-mediated knockout of the *BmC/EBPZ* gene. **(A)** Schematic diagram of the sgRNA-target site in the *BmC/EBPZ* gene. The boxes represent exons, the black sequences represent sgRNA sequences, and the red sequences represent the protospacer adjacent motif sequences. **(B)** Sequencing results of sgRNA recombinant plasmid. The red sequences represent two sgRNA sequences. **(C)** Phenotype of the *BmC/EBPZ* knockout strains. Larvae under natural light (left), larvae under red light (middle) and larvae under green light (right). Larvae that can express red and green fluorescence proteins are the *BmC/EBPZ* knockout strains (red arrows). **(D)** Various deletion genotypes in the *BmC/EBPZ* knockout strains. WT represents wild-type *898WB*. Δ1, Δ2, and Δ3 represent the three different knockout strains.

To verify whether the *BmC/EBPZ* gene was the major gene responsible for *GMS* mutation, we knocked out the expression of the *BmC/EBPZ* gene in the *Nistari* strain with the CRISPR/Cas9 system. We designed sgRNA 1 and sgRNA 2 on exons 8 and 10 in the *BmC/EBPZ* gene, respectively (Figure 3A). Two pairs of primers, each with two sgRNA site sequences, were synthesized, and the primers were named sgRNA1F, sgRNA1R, sgRNA2F, and sgRNA2R (Supplementary Table S4). We used the plasmids constructed in the laboratory and the above two pairs of primers for PCR amplification and recovered the amplified fragments. We used two amplified fragments and the initial plasmid to obtain a transgenic plasmid (pXL [IE1-DsRed-U6-sgRNA]) containing two sgRNA sites through homologous recombination (Supplementary Figure S2). The schematic diagram of how to connect two sgRNAs in one plasmid is shown in Supplementary Figure S3. The sequencing result of the recombinant plasmid is shown in Figure 3B. The primers were named V-F and V-R (Supplementary Table S4). We prepared a mixed injection containing recombinant plasmid (400 ng/μL) and *PiggyBac* helper plasmid (200 ng/μL), and injected 300 eggs. A total of 300 *Nistari* silkworm eggs were injected, 51% of the eggs successfully hatched into ant-silkworms, and 80% of the ant-silkworms survived to adults. The proportion of red fluorescent transgenic silkworms was 20%. The sgRNA transgenic silkworms expressing the red fluorescent protein was mated with the Cas9 transgenic silkworm expressing the green fluorescent protein to produce the *BmC/EBPZ* knockout strains. We obtained 11 moth batches of eggs that hatched into G_2_ generation mutants. Two moth batches of eggs were unfertilized, and nine moth batches of eggs were fertilized. The proportion of the fertilized eggs expressing double fluorescence was 25%. Compared with the control silkworms, all the knockout strains had small larvae, and the larvae died before the third instar. The larvae in the knockout strains varied in body size (Figure 3C). To confirm whether the above phenotypes were caused by the knockout of *BmC/EBPZ* gene, we extracted the genomic DNA of the *BmC/EBPZ* knockout strains and verified it by sequencing. Compared with the control group, the *BmC/EBPZ* knockout strains had different degrees of variation in genomic DNA (Figure 3D). This result confirmed that the *BmC/EBPZ* gene had been successfully knocked out in the *BmC/EBPZ* knockout strains. We performed qRT-PCR analysis on the three genes of *HSP70*, *HSP19.9* and *neutral alpha-glucosidase*. The results showed that the above three genes were differentially expressed between the *BmC/EBPZ* gene knockout strain and the wild-type *898WB*. In the *BmC/EBPZ* gene knockout strain, the expression levels of *HSP70* and *HSP19.9* genes were upregulated, while the expression levels of *neutral alpha-glucosidase* gene were downregulated (Figure 2D).

### Structure and phylogenetic analysis of the *BmC/EBPZ* gene

**FIGURE 4:**
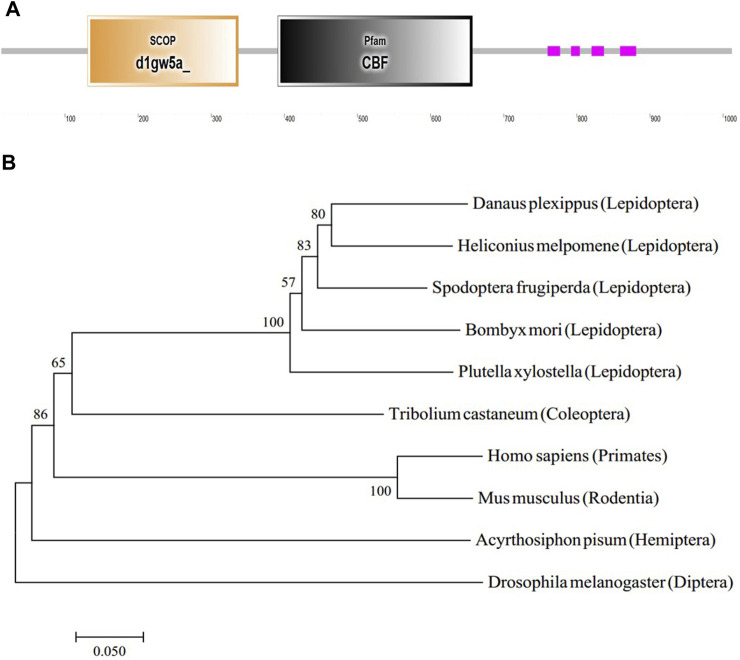
Structure and phylogenetic relationship of the *BmC/EBPZ* gene. **(A)** The predicted gene structure of the *BmC/EBPZ* gene. **(B)** Phylogenetic tree of the *BmC/EBPZ* gene in silkworms and other species.

The *GMS* mutant was caused by the 9 bp insertion in the *BmC/EBPZ* gene. To predict the function of the *BmC/EBPZ* gene in silkworms, we analyzed the protein domain with the SMART database. The *BmC/EBPZ* gene contained a CBF domain, which was present in the C/EBPs (Figure 4A). The two sgRNA sites were within the CBF domain of the *BmC/EBPZ* gene. The phylogenetic tree analysis of amino acid sequences from different species showed that the evolutionary relationship between silkworm and lepidoptera pests were close (Figure 4B).

## Discussion

The *GMS* mutation is spontaneous and occurs during mass rearing for silk production. We explored the molecular mechanism underlying this mutation by RNA-Seq (Mei et al., 2022). By conducting positional cloning and using a CRISPR/Cas9 system, we found that the *BmC/EBPZ* gene had a relatively high expression level in the testis. The deletion of the gene affected the growth and development of the silkworms. These results suggested that the *BmC/EBPZ* gene is the major gene responsible for *GMS* mutation.

C/EBPZ, the human homolog, belongs to the C/EBP family. In acute myeloid leukemia, C/EBPZ binds to the transcriptional initiation site of the *METTL3* gene and may play an important role in inflammatory response and cell differentiation (Liu et al., 2020). The genome sequences of patients with acute myeloid leukemia and gastric cancer were studied. Some of the patients carried *C/EBPZ* gene mutations, suggesting that the *C/EBPZ* gene can be used as a diagnosis or prognosis biomarker for the two types of cancer (Herold et al., 2014; Kori and Gov, 2022). Studies on the other members of the C/EBP family, such as C/EBP β and C/EBP δ, have shown that these transcription factors regulate the expression levels of genes involved in immune and inflammatory responses (Pérez-Pulido et al., 2021; Spek et al., 2021). The tissue-specific expression element C/EBP is located in the promoter of the NF-kb sequence and plays an important role in insects’ innate immunity (Liu et al., 2017). Moreover, C/EBP plays a key regulatory role in insect growth and development by enhancing histone acetylation. In silkworms, 20-hydroxyecdysone enhances the expression of the *BmC/EBP* gene through its receptor, thereby activating the expression of *BmCBP* gene, which then enhances histone H3K27 acetylation (Lyu et al., 2020).

KEGG pathway analysis of differential genes between the *GMS* mutant and *898WB* showed that the significantly enriched pathways were metabolic pathways and ECM-receptor interaction (Mei et al., 2022). C/EBP-2, a homolog of C/EBPs, affects fat storage in *C. elegans* by regulating the expression of key enzymes in the energy metabolism pathway, and C/EBP-2 deficiency has been observed in *Caenorhabditis elegans* with a low degree of lipid accumulation (Xu et al., 2015). Lipogenesis is regulated by central cascade transcription factors, including CEBPs (Xiong et al., 2021). A transcriptome data analysis of glioma patients showed that the ECM–receptor interaction is the most significant pathway in the KEGG pathway and the number of genes that directly interacted with C/EBP in the differential co-regulation network is extremely large, indicating that CEBP plays an essential role in glioma (Aouacheria et al., 2006). C/EBPZ containing a CBF domain is essential for growth and 60S ribosomal subunit biogenesis (Edskes et al., 1998). Other proteins containing this domain stimulate transcription from the HSP70 promoter (Edskes et al., 1998). C/EBPZ can regulate cell growth and differentiation and is highly tissue specific (O'Rourke et al., 1997). In this study, we successfully constructed the *BmC/EBPZ* gene knockout strain. After the *BmC/EBPZ* gene was knocked out, we found that the expression levels of heat shock signal transduction pathway related genes *HSP70* and *HSP19.9* were significantly upregulated, while the expression level of glycogen metabolism pathway related gene *neutral alpha-glucosidase* was significantly downregulated. The results suggest that the *BmC/EBPZ* gene may be involved in the regulation of heat shock signal transduction pathway and glycogen metabolism pathway.

The two sgRNA sites designed in this study were found within the CBF domain of the *BmC/EBPZ* gene and resulted in various degrees of deletion in the *BmC/EBPZ* gene of the *BmC/EBPZ* knockout strains, leading to the loss of function of the *BmC/EBPZ* gene. As mentioned above, the *BmC/EBPZ* gene was essential for the growth and development of organisms. The deletion of the coding sequence of the *BmC/EBPZ* gene should be the main factor of small larvae and even lethal of the knocked out strains. However, a 9 bp insertion was found in the coding sequence of the 17th exon (non-CBF domain) of the *BmC/EBPZ* gene in the *GMS* mutant. Compared with the gene deletion of the *BmC/EBPZ* knockout strains, the insertion of the short fragment in the nonfunctional domain should have less functional impact on the *BmC/EBPZ* gene. Therefore, the *GMS* mutant showed small larvae, whereas the *BmC/EBPZ* knockout strains were lethal in addition to small larvae. These results demonstrated that the *BmC/EBPZ* gene regulated silkworm growth.

We also studied the relative expression level of the *BmC/EBPZ* gene in different tissues. The gene was highly expressed in the testis and ovary. This results was consistent with the results of (Yokoi et al., 2021). The RT-qPCR results suggested that the *BmC/EBPZ* gene is involved in the regulation of reproduction-related genes. The phylogenetic tree of the *C/EBPZ* gene of different species showed that the silkworms have a close evolutionary relationship with other Lepidoptera insects such as *plutella xylostella* and *spodoptera frugiperda*. Therefore, the study of the *BmC/EBPZ* gene of silkworms can facilitates the development of novel technologies for controlling Lepidoptera pests.

## Data Availability

The coding sequence of BmC/EBPZ gene of the GMS mutant has been submitted to GenBank with the accession number, OR751402.1(https://www.ncbi.nlm.nih.gov/genbank/).

## Supplementary material

SUPPLEMENTARY FIGURE 1 Comparison of body size between GMS mutant and wild-type 898WB.

SUPPLEMENTARY FIGURE 2 Structure of the transgenic plasmid (pXL [IE1-DsRed-U6-sgRNA]).

SUPPLEMENTARY FIGURE 3 The schematic diagram of how to connect two sgRNAs in one plasmid.
