# Supplementary figures and images for: BmC/EBPZ gene is essential for the larval growth and development of silkworm, Bombyx mori
Source: Front Physiol. 2024 Mar 7;15:1298869. doi: 10.3389/fphys.2024.1298869 (PMC10959570; doi:10.3389/fphys.2024.1298869)

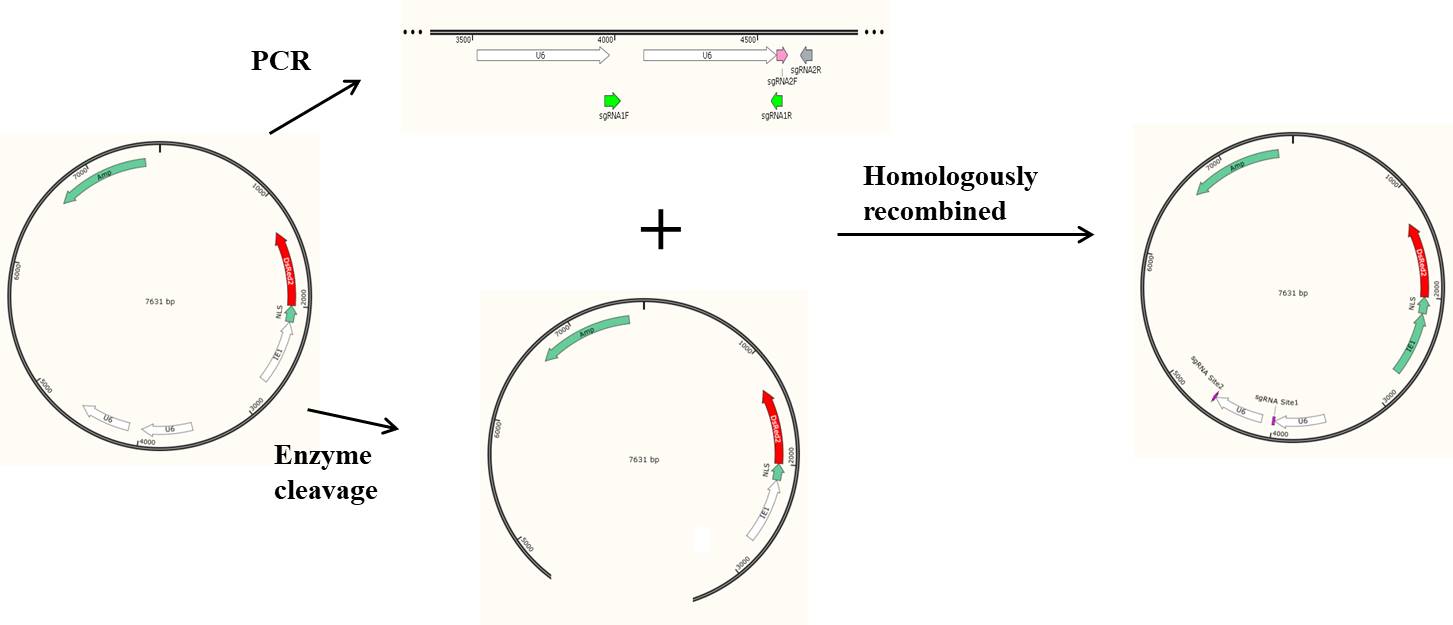

Supplement: Supplementary file 1 [file Image3.jpeg]

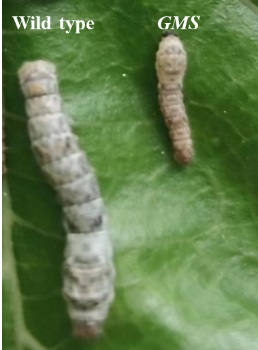

Supplement: Supplementary file 3 [file Image1.jpeg]

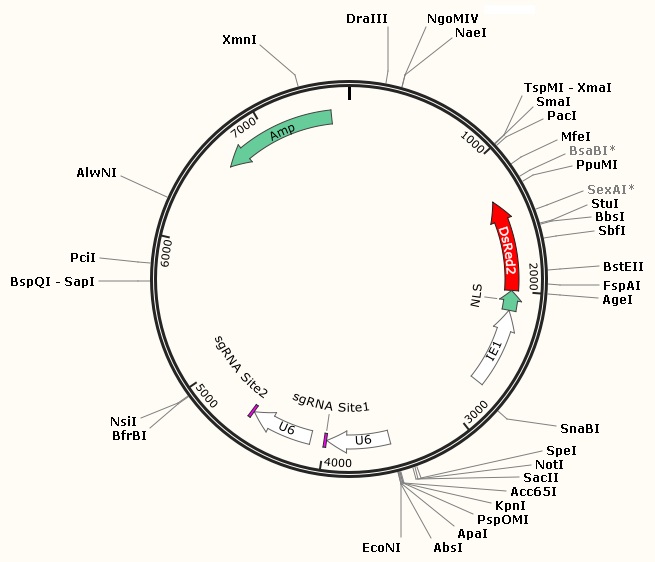

Supplement: Supplementary file 4 [file Image2.jpeg]
