# Supplementary material for: BmC/EBPZ gene is essential for the larval growth and development of silkworm, Bombyx mori
Source: Front Physiol. 2024 Mar 7;15:1298869. doi: 10.3389/fphys.2024.1298869 (PMC10959570; doi:10.3389/fphys.2024.1298869)
Supplement: Supplementary file 2 [file Table1.DOCX]

**Table S1. The polymorphic molecular genetic markers on chr24 of silkworm**

| **Number** | **Location on the chr24** | **Primer sequences (5´-3´)** | **Exchange number /** |
| --- | --- | --- | --- |
|  |  |  | **Total number of BC1M** |
| **chr24-71-7** | 4321056-4321077 | TGCAACGTTTTCCCAGTATTAT | 7/252 |
|  | 4321250-4321229 | AAAACCGCCAATTATTGTACTT |  |
| **chr24-71-12** | 4741810-4741831 | TTGATGATGCTATGAATCTTGG | 5/252 |
|  | 4743457-4743439 | CACGGCTGCTCATTCTGTT |  |
| **chr24-HinfI-3** | 5740920-5740940 | CACGGATTATGAAACCTACGA | 3/252 |
|  | 5741619-5741602 | ACCAGACGAGACTGCCGA |  |
| **chr24-39** | 6150706-6150727 | TCTGTTACTATCGTCCCCTGTC | 1/252 |
|  | 6150962-6150944 | TCGTCCACCCACTTATGCT |  |
| **chr24-53** | 6302869-6302888 | GTAGGTAAGCACGGGTAGGA | 1/252 |
|  | 6303114-6303096 | GTCTGAGGGCAAGGTAAGG |  |
| **chr24-43-2** | 7103573-7103552 | ACCGCCTATTGTTAGTGTCA | 1/252 |
|  | 7103245-7103264 | AATTCATAAGGTCGTGTCCTAC |  |
| **chr24-17** | 7321251-7321271 | CTGGTCTTAGGTGGTTACGAG | 3/252 |
|  | 7321330-7321312 | CTTGAGGGGTGAGATACGC |  |
| **chr24-75-1** | 15422883-15422864 | TTCGCGGTATCAACAGTCTC | 12/252 |
|  | 15422183-15422202 | TGTTGTAAGTTGGCAAGTGG |  |
