# Supplementary material for: BmC/EBPZ gene is essential for the larval growth and development of silkworm, Bombyx mori
Source: Front Physiol. 2024 Mar 7;15:1298869. doi: 10.3389/fphys.2024.1298869 (PMC10959570; doi:10.3389/fphys.2024.1298869)
Supplement: Supplementary file 5 [file Table2.DOCX]

| **ID** | **Location on the chr24** | **Description** |
| --- | --- | --- |
| **KWMTBOMO14436** | 6349586-6351077 | beclin-1-like protein |
| **KWMTBOMO14437** | 6356085-6376467 | uncharacterized protein LOC114241121 |
| **KWMTBOMO14438** | 6412645-6431087 | lysosome-associated membrane glycoprotein 1 |
| **KWMTBOMO14439** | 6434338-6448145 | uncharacterized protein LOC101739009 |
| **KWMTBOMO14440** | 6450638-6490222 | actin-depolymerizing factor 1 |
| **KWMTBOMO14441** | 6496330-6504237 | uncharacterized protein LOC101746134 |
| **KWMTBOMO14442** | 6505048-6512949 | leucine-rich repeat protein SHOC-2 isoform X1 |
| **KWMTBOMO14443** | 6519959-6535727 | seipin isoform X1 |
| **KWMTBOMO14444** | 6537068-6541485 | valine--tRNA ligase |
| **KWMTBOMO14445** | 6546912-6548447 | uncharacterized protein LOC101739492 |
| **KWMTBOMO14446** | 6550341-6554867 | LRP16 protein |
| **KWMTBOMO14447** | 6555888-6582534 | neutral alpha-glucosidase AB-like isoform X2 |
| **KWMTBOMO14448** | 6587549-6594747 | zinc finger protein 59-like |
| **KWMTBOMO14449** | 6597588-6606407 | zinc finger protein 343 isoform X1 |
| **KWMTBOMO14450** | 6613529-6620327 | poly(A) polymerase type 3 |
| **KWMTBOMO14451** | 6693566-6699534 | reverse transcriptase |
| **KWMTBOMO14452** | 6718905-6719524 | serine/threonine-protein kinase MARK2 isoform X9 |
| **KWMTBOMO14453** | 6770655-6771856 | MAP/microtubule affinity-regulating kinase 3-like |
| **KWMTBOMO14454** | 6812745-6853182 | MAP/microtubule affinity-regulating kinase 3-like |
| **KWMTBOMO14455** | 6862436-6868444 | putative cuticle protein CPH35 |
| **KWMTBOMO14456** | 6909255-6910508 | deoxyhypusine synthase |
| **KWMTBOMO14457** | 6911405-6956689 | tyrosine-protein kinase-like otk |
| **KWMTBOMO14458** | 6965205-6976114 | actin-related protein 2 |
| **KWMTBOMO14459** | 6976505-6996629 | CCAAT/enhancer-binding protein zeta |
| **KWMTBOMO14461** | 7002415-7003089 | ribosomal protein S6 kinase alpha-5 isoform X1 |
| **KWMTBOMO14462** | 7013895-7074132 | ribosomal protein S6 kinase alpha-4-like isoform X2 |
| **KWMTBOMO14463** | 7084626-7090364 | Pol protein |

**Table S2.** **The candidate genes mapped on the chr24 of silkworm**

The red marks represent the *BmC/EBPZ* gene.
