# Supplementary material for: BmC/EBPZ gene is essential for the larval growth and development of silkworm, Bombyx mori
Source: Front Physiol. 2024 Mar 7;15:1298869. doi: 10.3389/fphys.2024.1298869 (PMC10959570; doi:10.3389/fphys.2024.1298869)
Supplement: Supplementary file 6 [file Table3.DOCX]

**Table S3. Primers used for amplifying cDNA of the *BmC/EBPZ* gene**

| **Primer name** | **Primer sequences (5´-3´)** | |
| --- | --- | --- |
| CCAATF | | CTCAGTTCTCTGTTTGCGTCA |
| CCAATR | | TTCCCACCAGGTCCGTTC |
| HSP70F  HSP70R  HSP19.9F  HSP19.9R  NAGF  NAGR  GAPDHF | | AGATGGGAGGGTTTGCTATGT  CATTGTGCTGGATGAACTGCT  GCAAACACGAGGAGAAGAAAGA  TGGGTGCGATTACAGACAACA  ATAATAGGCAACCGGACAATA  GATCTAATAAGAACCCGCACAA  ACATCATTCCTGCCTCTACTG |
| GAPDHR | | CCTCCTTGACCTTTTGCTT |
