# Supplementary material for: BmC/EBPZ gene is essential for the larval growth and development of silkworm, Bombyx mori
Source: Front Physiol. 2024 Mar 7;15:1298869. doi: 10.3389/fphys.2024.1298869 (PMC10959570; doi:10.3389/fphys.2024.1298869)
Supplement: Supplementary file 7 [file Table4.DOCX]

**Table S4. Primers used to construct transgenic plasmid**

| **Primer name** | **Primer sequences (5´-3´)** | |
| --- | --- | --- |
| **sgRNA1F** | | AATATCGTGCTCTACAAGTGGACAAGAACATTCCCAGGGGTTTTA  GAGCTAGAAATAGC |
| **sgRNA1R** | | CGCCGTCGATTTTAAGATCCACTTGTAGAGCACGATATT |
| **sgRNA2F** | | GATCTTAAAATCGACGGCGGTTTTAGAGCTAGAAATAGC |
| **sgRNA2R** | | TATAGATATCAAGCTGCTAGAAAAAAAAGCACCGACTCGG |
| **V-F** | | GAAAAAAAAAGCACCGACTCGG |
| **V-R** | | GTGGAGCTCCAGCTTTTGTTC |

The red marks represent two sgRNA target sequences, respectively.

The underline marks represent the reverse complement sequence of sgRNA 2.
